# Efficacy of Tigecycline and Linezolid Against Pan-Drug-Resistant Bacteria Isolated From Companion Dogs in South Korea

**DOI:** 10.3389/fvets.2021.693506

**Published:** 2021-08-06

**Authors:** Dong-Hyun Kim, Jung-Hyun Kim

**Affiliations:** Department of Veterinary Internal Medicine, College of Veterinary Medicine, Konkuk University, Seoul, South Korea

**Keywords:** linezolid, tigecycline, antimicrobial resistance, pan-drug-resistant, companion animals

## Abstract

The emergence of multidrug-resistant bacteria in companion animals is an increasing concern in view of the concept of One Health. The antimicrobials linezolid (LZD) and tigecycline (TGC) are effective against multidrug-resistant bacteria isolated from humans; however, thus far, no previous study has evaluated the efficacy of these drugs against bacteria isolated from companion animals. This study aimed to evaluate the efficacy of LZD and TGC against bacteria that were isolated from companion dogs and showed resistance to all classes of antimicrobial agents. Clinical samples (auditory channel, eye, skin, and urine) were collected from dogs that visited the Veterinary Medical Teaching Hospital of Konkuk University (Seoul, South Korea) from October 2017 to September 2020. In total, 392 bacterial isolates were obtained, of which 85 were resistant to all classes of antimicrobial agents tested and were, therefore, considered potentially pan-drug resistant (PDR). The susceptibility of isolates to LZD and TGC was determined by the disk diffusion method and interpreted using the Clinical Laboratory Standards Institute guidelines. In total, 95.6% (43/45) and 97.8% (44/45) of gram-positive isolates were susceptible to LZD and TGC, respectively, whereas 82.5% (33/40) of gram-negative isolates were sensitive to TGC. In conclusion, both agents showed favorable efficacy, with the susceptibility rates for all potential PDR bacteria, except *Pseudomonas* spp., ranging from 72.7 to 100%. Thus, these drugs may serve as excellent antimicrobial options for veterinary medicine in the future.

## Introduction

The emergence of multidrug-resistant (MDR) bacteria, which are resistant to three or more categories of antimicrobials, in companion animals is highly concerning. Particularly, resistance to antimicrobials is growing among bacteria such as *Staphylococcus aureus, Staphylococcus pseudintermedius*, and *Escherichia coli* (1), which cause infections in dogs. The transmission of such bacteria can be either direct or indirect among dogs, owners, and veterinary staff. Indeed, practicing veterinarians are far more likely to experience nasal colonization with *S. aureus* than the general population (2). Notably, under the One Health concept, companion animals have been documented to be reservoirs of some high-risk MDR clones of Enterobacteriaceae (3), which are likely to be acquired from their human owners. Overuse of antimicrobials in veterinary clinics may amplify antimicrobial resistance and result in a subsequent spread of resistant microorganisms to animal owners. Therefore, the appropriate use of antimicrobials to prevent pain, illness, or death should be adopted for infection management in animals to improve the public health of both humans and animals.

Previous studies that analyzed the temporal trends of antimicrobial resistance in small collections of bacterial isolates from infected companion animals have provided evidence for a significant increase in antimicrobial resistance, particularly to agents frequently used in clinical settings, such as cephalosporins, ampicillins, and quinolones (4).

Linezolid (LZD) is a member of the oxazolidinone class of synthetic antibacterial agents that inhibit bacterial protein synthesis through a unique mechanism. In contrast to other inhibitors of protein synthesis, oxazolidinones act early in translation by preventing the formation of a functional initiation complex (5). Discovered in 1987 at E.I. DuPont de Nemours and Co., Inc., DuP-721 was the first well-characterized oxazolidinone (5) that exhibited strong activity against MDR gram-positive pathogens (6). In fact, it is currently used for the treatment of clinical methicillin-susceptible and methicillin-resistant *S. aureus* infections and for managing infections caused by vancomycin-resistant enterococci (7) in humans.

Tigecycline (TGC) is a glycylcycline antibiotic that is effective against a variety of gram-positive and gram-negative bacteria (8). TGC is currently one of the most potent antimicrobial agents for treating infections caused by MDR bacteria in humans (8). This drug has demonstrated *in vitro* activity against important resistant organisms, including methicillin-resistant *S. aureus*, penicillin-resistant *Streptococcus pneumoniae*, and vancomycin-resistant enterococcal species, in addition to extended-spectrum beta-lactamase-producing *E. coli* and *Klebsiella pneumoniae* (9). A previous study has shown that more than 90% of Enterobacteriaceae isolates are susceptible to this drug (10).

Nevertheless, LZD and TGC have not been used as first- or second-line treatment options in companion animals owing to concerns that the overuse and abuse of antimicrobials in animals would limit treatment options for human bacterial infections, in view of the One Health concept. Moreover, no previous study has evaluated the efficacies of LZD and TGC against bacteria originating from companion animals.

The present study aimed to evaluate the efficacies of LZD and TGC against potential pan-drug-resistant (PDR) bacteria (i.e., resistant to all classes of antimicrobial agents) isolated from dogs. Antimicrobial stewardship and related policies are beyond the scope of the current work.

## Materials and Methods

### Sampling

A total of 359 clinical samples were collected from different lesions in dogs that visited the Veterinary Medical Teaching Hospital of Konkuk University (Seoul, South Korea) from October 2017 to September 2020. The samples were immediately placed into a transport medium (ESwab, Copan, Brescia, Italy). The sampling sites included the auditory channels, eyes/conjunctiva, gastrointestinal tract, skin/mucosa, blood, and urogenital tract (Table 1). Auditory channel, eye, and skin samples were routinely collected using sterile cotton swabs, and urine samples were collected by cystocentesis. In addition, we collected at least 2 g of feces, which were cubed to ~$\frac{1}{2}$ to $\frac{3}{4}$ inch on a single side using a fecal loop. Peripheral blood was collected by venipuncture of the jugular vein to identify systemic infections. Furthermore, cerebrospinal fluid was obtained at the junction between lumbar vertebrae 5 and 6 using a conventional lumbar tapping method, and samples from the peritoneal walls were collected with sterile cotton swabs. All samples were immediately transported to the NosVet Laboratory (Gyeonggi-do, South Korea) and analyzed within 3–4 h.

**Table 1:** Distribution of sampling sites and isolates.

**Sites**	**No. of samples**	**No. of isolates**
Urogenital tract	101	99
Auditory channel	92	80
Skin/mucosa	79	105
Eye/conjunctiva	33	30
Gastrointestinal tract	10	25
Tooth	7	7
Joint effusion	7	0
Blood	6	5
Respiratory tract	5	17
Other sites^*^	19	24
Total	359	392

* *Other sites include cerebrospinal fluid, tissue of various types, synovial capsule, foreign body, mass, bone (Supplementary File 1)*.

The animal study and the protocol was reviewed and approved by the Institutional Animal Care and Use Committee (KU20218). Written informed consent was obtained from the owners for the participation of their animals in this study.

### Bacterial Isolates

In total, 392 isolates were obtained from the dogs by directly inoculating blood agar plates with the clinical samples using cotton swabs, followed by incubation of the agar plates at 37°C for up to 24 h. Morphologically identical colonies were picked and sub-cultured onto blood agar plates, and species were identified using a matrix-assisted laser desorption/ionization mass spectrometer (ASTA, Gyeonggi-do, South Korea). Bacterial stock solutions were stored at −20°C.

### Antibiotic Susceptibility Testing

Commercial antimicrobial disk diffusion tests were performed by NosVet, Inc., according to the Clinical Laboratory Standards Institute (CLSI) guidelines (VET08). Susceptibility to 21 antibiotics from 10 classes, namely, amikacin (AK), amoxicillin/clavulanic acid (AMC), ampicillin (AMP), azithromycin (AZM), cefixime (CFM), cefotaxime (CTX), cefpodoxime (CPD), ceftazidime (CAZ), cephalexin (CL), cephazolin (KZ), ciprofloxacin (CIP), clindamycin (DA), doxycycline (DO), enrofloxacin (ENR), erythromycin (E), gentamicin (CN), lincomycin (MY), ofloxacin (OFX), spiramycin (SP), sulfamethoxazole/trimethoprim (SXT), and tetracycline (TE), was determined using a Vitek® AST-P601 card (bioMérieux, Marcy l'Étoile, France).

Two additional antibiotics, namely, LZD and TGC (Thermo Fisher Scientific, Waltham, MA, USA), which are not included in the CLSI VET08 guidelines, were tested against potential PDR strains. Susceptibility to LZD and TGC was determined by the disk diffusion method and interpreted based on the CLSI guidelines. The European Committee on Antimicrobial Susceptibility Testing (EUCAST) guidelines were used when information was missing in the CLSI guidelines. Briefly, bacteria were inoculated from stock solutions onto Mueller–Hinton agar plates and incubated at 37°C for 24 h. Colonies were suspended in normal saline, and the turbidity was adjusted to a 0.5 McFarland standard equivalent (~10^8^ colony-forming units per milliliter). Sterile cotton swabs were dipped into inoculation broth and subsequently streaked over Mueller–Hinton agar plates. Antibiotic disks of 30 μg of LZD and 15 μg of TGC were then placed on these plates, followed by incubation of the plates at 37°C for 24 h. Diameters of the inhibition zones were used to categorize bacteria as susceptible, intermediate resistant, and resistant according to the CLSI and EUCAST guidelines.

### Statistical Analysis

Descriptive statistics were used for the analyses of signalment, clinical data, and laboratory findings. All statistical analyses were performed using Microsoft Excel (Microsoft Corp., Redmond, WA, USA). An exact chi-square test was used to compare the efficacies of LZD, TGC, and the 21 other antibiotics against potential PDR bacteria from dogs. Differences with *P*-values of <0.05 were considered statistically significant.

## Results

Based on the antibiotic sensitivity evaluation conducted by NosVet, Inc., 211 bacterial isolates were classified as extensively drug resistant (XDR), as previously described (11). Of these 211 isolates, 57 were resistant to eight classes and 69 were resistant to nine classes, while 85 were resistant to all ten classes of antimicrobial agents tested and were thus considered potential PDR bacteria (11). Table 2 presents the taxonomic distribution of the 85 isolates.

**Table 2:** Species distribution of potential pan-drug-resistant isolates tested in this study.

**Gram**	**Species**	**No. of isolates**
Negative	*Escherichia coli*	12
	*Klebsiella pneumoniae*	11
	*Proteus mirabilis*	10
	*Pseudomonas aeruginosa*	3
	*Citrobacter freundii*	1
	*Enterobacter aerogenes*	1
	*Enterobacter cloacae complex*	1
	Pasteurellaceae bacterium	1
Positive	*Staphylococcus pseudintermedius*	26
	*Enterococcus faecium*	6
	*Enterococcus faecalis*	5
	*Staphylococcus schleiferi*	3
	*Corynebacterium auriscanis*	2
	*Rothia nasimurium*	1
	*Staphylococcus epidermidis*	1
	*Streptococcus canis*	1
Total		85

### Gram-Positive Isolates

According to the tests conducted by NosVet, Inc., 163 (65%) of 249 gram-positive strains showed sensitivity to AK, 131 (52.6%) were sensitive to AMC, 90 (36.1%) were sensitive to AMP, 110 (44.2%) were sensitive to AZM, 5 (2.0%) were sensitive to CFM, 89 (35.7%) were sensitive to CTX, 88 (35.3%) were sensitive to CPD, 11 (4.4%) were sensitive to CAZ, 97 (39.0%) were sensitive to CL, 114 (45.8%) were sensitive to KZ, 20 (8.0%) were sensitive to CIP, 93 (37.3%) were sensitive to DA, 124 (49.8%) were sensitive to DO, 133 (53.4%) were sensitive to ENR, 99 (39.8%) were sensitive to E, 88 (35.3%) were sensitive to CN, 8 (3.2%) were sensitive to MY, 125 (50.2%) were sensitive to OFX, 87 (34.9%) were sensitive to SP, 84 (33.7%) were sensitive to SXT, and 85 (34.1%) were sensitive to TE. Among these strains, 215 (86.3%) were MDR, 125 (50.2%) were XDR, and 45 (18.0%) were potential PDR bacteria (Supplementary File 1).

**Figure 1:**
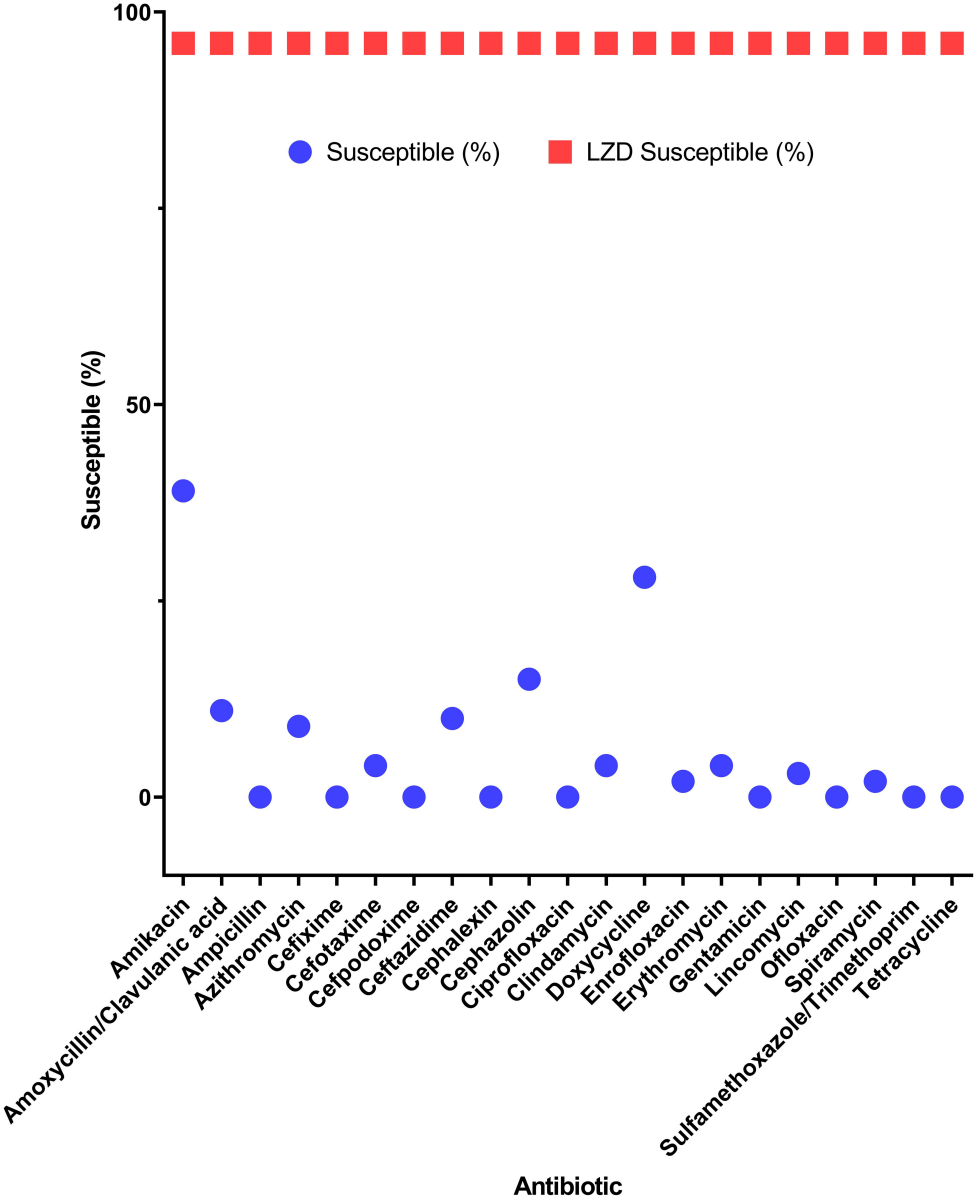
Efficacy of linezolid compared to 21 antibiotics for potential pan-drug-resistant (PDR) bacteria from dogs. Circles indicate the susceptibility of gram-positive potential PDR isolates for each antibiotic. Squares indicate the average susceptibility of gram-positive potential PDR isolates.

**Figure 2:**
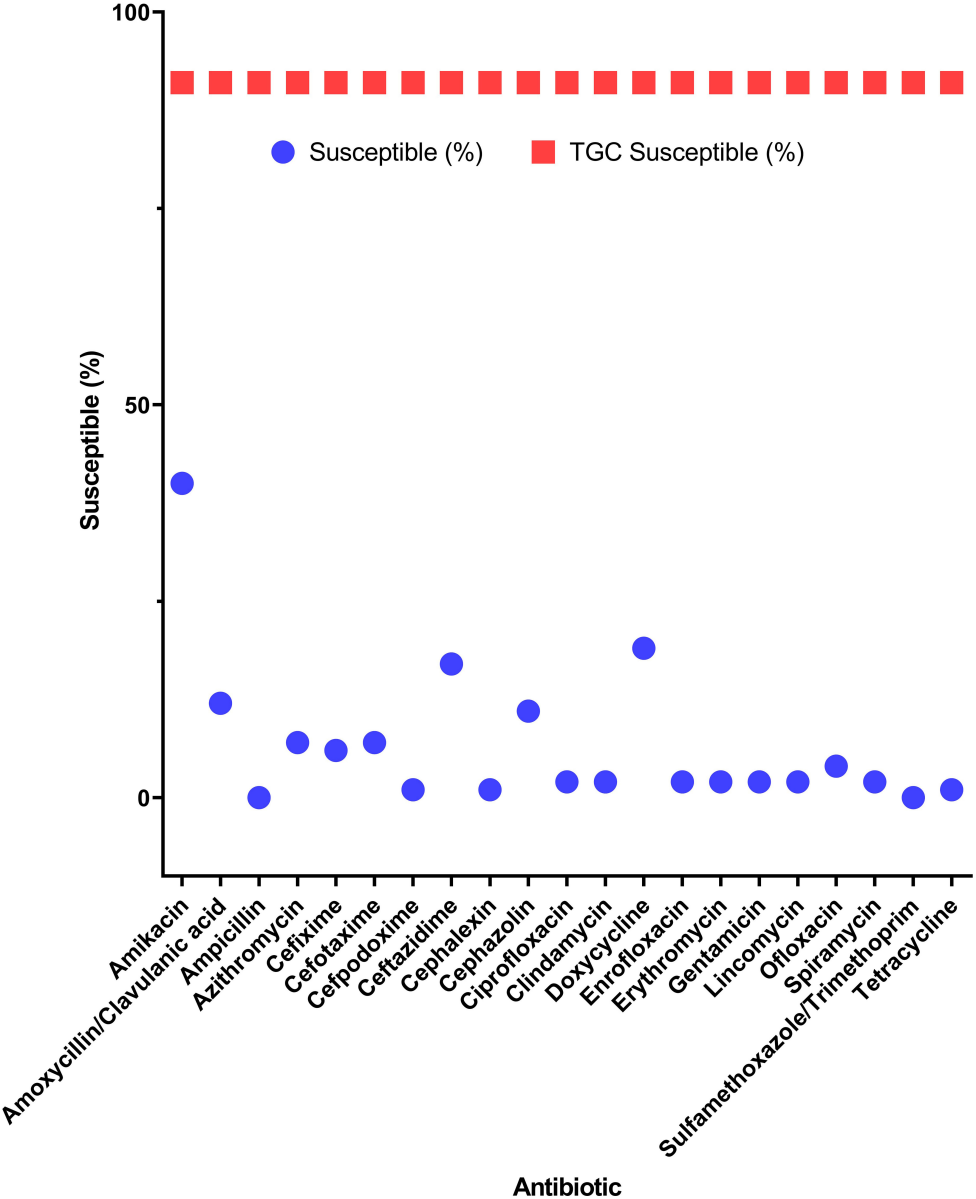
Efficacy of tigecycline compared to 21 antibiotics for potential pan-drug-resistant (PDR) bacteria from dogs. Circles indicate the susceptibility of potential PDR isolates for each antibiotic. Squares indicate the average susceptibility of potential PDR isolates.

Among the potential PDR gram-positive bacteria, 95.6% (43/45) and 97.8% (44/45) of isolates were susceptible to LZD (Figure 1) and TGC (Figure 2), respectively. Superiority of LZD and TGC over the other antibiotics was statistically analyzed, and the result is presented in Tables 3, 4. Overall, the potential PDR bacteria were significantly more susceptible to these two agents than to the other 21 agents (*P* < 0.05). The average diameter of the zone of inhibition for LZD was 31 mm, which fell within the susceptibility zone diameter of gram-positive bacteria for LZD. The average diameter of the zone of inhibition was 24.5 mm for TGC, which exceeded the zone of resistance size.

**Table 3:** Superiority of linezolid efficacy over 21 different antibiotics to eradicate potential pan-drug-resistant bacteria from dogs.

**Antibiotic**	**Susceptible (%)**	**Linezolid *P-value***
Amikacin	39.1%	<0.001
Amoxycillin/Clavulanic acid	10.9%	<0.001
Ampicillin	0.0%	<0.001
Azithromycin	8.7%	<0.001
Cefixime	0.0%	<0.001
Cefotaxime	4.3%	<0.001
Cefpodoxime	0.0%	<0.001
Ceftazidime	9.7%	<0.001
Cephalexin	0.0%	<0.001
Cephazolin	15.2%	<0.001
Ciprofloxacin	0.0%	<0.001
Clindamycin	4.3%	<0.001
Doxycycline	28.3%	<0.001
Enrofloxacin	2.2%	<0.001
Erythromycin	4.3%	<0.001
Gentamicin	0.0%	<0.001
Lincomycin	3.2%	<0.001
Ofloxacin	0.0%	<0.001
Spiramycin	2.2%	<0.001
Sulfamethoxazole/Trimethoprim	0.0%	<0.001
Tetracycline	0.0%	<0.001
Linezolid	95.7%	

**Table 4:** Superiority of tigecycline efficacy over 21 different antibiotics to eradicate potential pan-drug-resistant bacteria from dogs.

**Antibiotic**	**Susceptible (%)**	**Tigecycline *P-value***
Amikacin	40.0%	<0.001
Amoxycillin/Clavulanic acid	11.8%	<0.001
Ampicillin	0.0%	<0.001
Azithromycin	7.1%	<0.001
Cefixime	5.7%	<0.001
Cefotaxime	7.1%	<0.001
Cefpodoxime	1.2%	<0.001
Ceftazidime	17.1%	<0.001
Cephalexin	1.2%	<0.001
Cephazolin	10.6%	<0.001
Ciprofloxacin	1.9%	<0.001
Clindamycin	2.4%	<0.001
Doxycycline	18.8%	<0.001
Enrofloxacin	2.4%	<0.001
Erythromycin	2.4%	<0.001
Gentamicin	2.4%	<0.001
Lincomycin	1.9%	<0.001
Ofloxacin	3.5%	<0.001
Spiramycin	1.5%	<0.001
Sulfamethoxazole/Trimethoprim	0.0%	<0.001
Tetracycline	1.2%	<0.001
Tigecycline	90.6%	

#### *Staphylococcus* spp.

One hundred sixty *Staphylococcus* spp. isolates showed sensitivity to AK (93.7%), followed by AMC (53.1%) and DA (52.4%). Of these isolates, 30 (18.7%) were potential PDR strains, and all of them were susceptible to LZD and TGC, with an average diameter of the zone of inhibition of 31.7 mm.

#### *Enterococcus* spp.

Forty-two *Enterococcus* spp. isolates showed sensitivity to AMP (17.5%), followed by AMC (16.8%). Of these isolates, 11 (26.2%) were potential PDR strains. The sensitivity of potential PDR *Enterococcus faecium* and *Enterococcus faecalis* isolates to LZD was 83.3% (5/6) and 80.0% (4/5), respectively, and the sensitivity to TGC was 83.3% (5/6) and 100% (5/5), respectively.

#### Uncommonly Encountered Species

One *Rothia nasimurium*, one *Streptococcus canis*, and two potential PDR *Corynebacterium auriscanis* isolates were susceptible to LZD and TGC.

### Gram-Negative Isolates

According to the tests conducted by NosVet, Inc., 105 (73.4%) of the 143 gram-negative strains showed sensitivity to AK, 68 (47.6%) were sensitive to AMC, 33 (23.1%) were sensitive to AMP, 37 (25.9%) were sensitive to AZM, 56 (39.2%) were sensitive to CFM, 61 (42.7%) were sensitive to CTX, 59 (41.3%) were sensitive to CPD, 83 (58.0%) were sensitive to CAZ, 55 (38.5%) were sensitive to CL, 49 (34.3%) were sensitive to KZ, 17 (11.9%) showed sensitivity to CIP, 4 (2.8%) were sensitive to DA, 58 (40.6%) were sensitive to DO, 64 (44.8%) were sensitive to ENR, 11 (7.7%) were sensitive to E, 79 (55.2%) were sensitive to CN, 1 (0.7%) showed sensitivity to MY, 71 (49.7%) were sensitive to OFX, 1 (0.7%) showed sensitivity to SP, 53 (37.1%) were sensitive to SXT, and 50 (35.0%) were sensitive to TE. Among these strains, 130 (90.9%) were MDR, 87 (60.8%) were XDR, and 40 (27.9%) were potential PDR bacteria (Supplementary File 1).

Of note, 82.5% (33/40) of the potential PDR gram-negative isolates were sensitive to TGC. The average inhibition zone diameter was 18.5 mm for positive isolates, which slightly exceeded the resistance cutoff. Among Enterobacteriaceae, 100% (12/12) of the *E. coli* strains and 72.7% (8/11) of the *K. pneumoniae* strains, as well as single *Citrobacter freundii, Enterobacter aerogenes*, and *Enterobacter cloacae* complex isolates, were susceptible to TGC. The overall sensitivity to TGC was 88.5% (23/26).

#### Escherichia coli

Fifty-two *E. coli* isolates showed sensitivities of 27.3% to AK and 21.0% to CN. Of these, 12 (23.0%) were potential PDR strains, as recommended by the CLSI VET08 guidelines. The average diameter of the zone of inhibition for TGC was 18.6 mm, which fell within the susceptibility zone (≥18 mm).

#### Klebsiella pneumoniae

Nineteen isolates of *K. pneumoniae* showed <10% sensitivity to the 21 antibiotics, and 11 (57.8%) were potential PDR strains, which was very high compared to that in other species. Furthermore, *K. pneumoniae* showed the highest resistance rate (27.3%; 3/11) to TGC among the bacteria tested in this study. The inhibition zone diameters of the resistant isolates were in the range of 13–17 mm, which was within the resistance zone (≥18 mm).

#### Proteus mirabilis

Among the 31 *P. mirabilis* isolates, 10 were classified as potential PDR strains, and only one of these (10%; 1/10) was classified as being resistant to TGC.

#### Pseudomonas aeruginosa

*P. aeruginosa* isolates were less sensitive to the 21 antibiotics tested than those of other species. Only three of the 13 isolates were classified as potential PDR strains, and all three isolates (100%) were resistant to TGC, with an inhibition zone diameter of 0 mm.

#### Uncommonly Encountered Species

One (100%) isolate of each of the following species was susceptible to TGC: *C. freundii, E. aerogenes, E. cloacae* complex, and an unclassified species of Pasteurellaceae.

## Discussion

To the best of our knowledge, no studies reported the susceptibility of *S. aureus*, isolated from animals, to LZD and TGC; however, some reports indicated that all human *S. aureus* isolates show susceptibility to these drugs (12, 13), consistent with our isolates from dog samples.

*S. pseudintermedius*, the most common opportunistic pathogen in dogs (14), exhibits resistance to commonly used antimicrobials (15). It is the most common pathogen causing recurrent skin infections in dogs because of allergies, endocrine diseases, or other immunocompromising factors including old age and cancer (15). The possibility of *S. pseudintermedius* transmission from animals to humans was evaluated in four clinical human cases, among which two dog owners and their dogs carried identical *S. pseudintermedius* strains (16). Although some strains can acquire methicillin resistance and cause severe refractory infections, even potential PDR strains are susceptible to LZD and TGC.

*R. nasimurium* and *S. pseudintermedius* can exhibit increased pathogenicity through synergistic effects (17); if these bacteria are potential PDR, then the associated fatality rate can significantly increase.

*E. faecalis* is the most frequently encountered enterococcal species in the anus and tonsils of dogs, followed by *E. faecium* (18). These two species are considered the third and fourth most prevalent nosocomial human pathogens worldwide, respectively (18), necessitating their control in both humans and animals. Although there is scarce evidence for susceptibility testing of *Enterococcus* spp. in animal samples, the sensitivity of *E. faecalis* and *E. faecium* isolates from human samples to LZD was 94.3 and 93.5%, respectively (12). In another study, the sensitivity of *Enterococcus* spp. isolates to TGC was 100% (201/201) (19), consistent with our results.

Oxazolidinones, including LZD, are excluded from gram-negative bacteria-related infection treatment because they enhance pump activity against LZD and expel the antibiotic from the cytoplasm, resulting in lower LZD accumulation levels in *E. coli, C. freundii*, and *E. aerogenes* than in *S. aureus* and *E. faecium* (20).

*E. coli* is frequently encountered and causes severe infections in both humans and dogs (21). The possible transmission of virulent and/or resistant *E. coli* strains between animals and humans through numerous pathways is highly concerning (21). *E. coli* represents a main reservoir of resistance genes probably responsible for treatment failure in both human and veterinary medicine (22). Indeed, an increasing number of resistance genes have been identified in *E. coli* during the past decades, mostly acquired through horizontal gene transfer (23). In the enterobacterial gene pool, *E. coli* acts as a donor and recipient of resistance genes from other bacteria (24). Hence, the broad-spectrum resistance of this species is quite likely, considering that most recommended antimicrobials do not effectively inhibit its growth. Therefore, TGC may broaden antimicrobial treatment choices for refractory *E. coli* infections.

*K. pneumoniae* is an important nosocomial agent that spreads easily (25) and causes community-onset infections in companion animals and humans. It is the second most common Enterobacteriaceae species causing urinary tract infections in dogs; strains are frequently MDR, posing important therapeutic limitations (26, 27). In previous studies, 84.6% (55/60) and 87.6% (340/388) of human *K. pneumoniae* isolates showed TGC susceptibility (19, 28). Our results showed a lower susceptibility TGC rate (73.7%), indicating the stronger resistance of isolates of animal origins to TGC. The resistance gene appears to have originated from the chromosome of a *Pseudomonas* species and may have been transferred to plasmids by adjacent site-specific integrases. Although the gene appears to be rare in human clinical isolates, the transferability of the gene cluster and its broad-spectrum substrate make further dissemination of this mobile TGC resistance determinant possible (29). The rapid development of TGC resistance necessitates further expansion of other treatment options.

Human *Proteus* spp. and *P. aeruginosa* isolates exhibit strong resistance to TGC. Nevertheless, we evaluated their susceptibility to TGC because strains of the same species may have different antimicrobial susceptibility profiles depending on their host of origin (30). *P. mirabilis* is the epitome of an opportunistic nosocomial pathogen in humans and animals (31), causing urinary tract infections (32) and chronic otitis externa (33) in companion animals. Moreover, *P. mirabilis* has low susceptibility to TGC (34). Moreover, a novel TGC resistance gene, *tet*, has recently been identified in *Proteus* species isolated from animals (35). Fortunately, isolates from the current study, collected over 3 years, showed sensitivity to TGC, suggesting that TGC-resistant *P. mirabilis* has not yet been disseminated in Korea.

*P. aeruginosa* is a clinically important opportunistic pathogen causing serious acute and chronic infections (36). It is ubiquitous in the environment and can persist in water and soil despite minimal nutrients, tolerating a broad spectrum of humidity and temperature conditions (37). *P. aeruginosa* is one of the pathogens that most frequently acquire or develop multidrug resistance (37). The exceptional array of intrinsic and acquired drug resistance mechanisms employed by *P. aeruginosa* renders the antibiotic-based treatment of these infections difficult. One important resistance mechanism is mediated by the resistance–nodulation–cell division family of efflux pumps (38). In one study, all (15/15) human *P. aeruginosa* isolates were resistant to TGC (28). Similarly, our results showed that TGC is not suitable for treating *P. aeruginosa* infections.

*C. auriscanis* was first discovered in a dog with ear infection (39) and typically acts as an opportunist in mixed infections associated with bacterial otitis externa, which can be resolved by treating and controlling other causative agents, but it may have pathological significance when occurring alone (40). *C. auriscanis* is often resistant to beta-lactam antibiotics; therefore, other antimicrobials may be necessary if skin lesions are not resolved after antimicrobial therapy (41).

*S. canis* is considered part of the healthy microbiota of the skin and mucosa of dogs but may be responsible for opportunistic infections. In dogs, *S. canis* is isolated from skin infections, urogenital and respiratory tract infections, otitis externa, septicemia, necrotizing fasciitis, and streptococcal toxic shock syndrome (42). Only one strain was tested in this study and found to be susceptible to LZD and TGC, indicating they are possible treatment options for *S. canis* infections.

*E. aerogenes* and *E. cloacae* complexes are members of the intestinal microbiota and are commonly MDR. Fortunately, both isolates were susceptible to TGC in this study, indicating the potential application of TGC for treating urinary tract infections, accounting for 38% (8/21) of animal cases, and wound infections, accounting for 19% (4/21) (43). *C. freundii* is intrinsically resistant to AMP, AMP/sulbactam, and cephalosporins and causes sepsis in dogs (44). A single isolate of this species was tested in this study and was susceptible to TGC; therefore, this antibiotic may be considered for treating *C. freundii* infections.

This study has several limitations. First, for certain bacteria (e.g., *C. auriscanis*, Pasteurellaceae, *P. mirabilis, P. aeruginosa*, and *R. nasimurium*), there are no susceptibility or resistance criteria. Second, many countries prohibit LZD and TGC use in animal settings to preserve treatment options for human infections. However, these agents may be employed in the future under well-organized antimicrobial stewardship frameworks and policies, and our results provide foundational knowledge for using LZD and TGC in veterinary medicine.

In conclusion, we evaluated the efficacies of TGC and LZD against potential PDR bacteria that are frequently isolated from companion dogs, and the results showed resistance of the organisms to all other antimicrobial classes recommended by the veterinary CLSI guidelines. Both agents showed favorable efficacy, with susceptibility rates of all potential PDR bacteria, except *P. aeruginosa*, ranging from 72.7 to 100%. Thus, TGC and LZD may serve as promising antimicrobial options for veterinary medicine in the future. For application in patients, *in vivo* pharmacokinetic and pharmacological studies are needed. To avoid exacerbating bacterial antibiotic resistance, legal regulations for TGC and LZD are needed to prevent their misuse.

## Data Availability Statement

The original contributions presented in the study are included in the article/Supplementary Material, further inquiries can be directed to the corresponding author/s.

## Ethics Statement

The animal study was reviewed and approved by Institutional Animal Care and Use Committee (KU20218). Written informed consent was obtained from the owners for the participation of their animals in this study.

## Author Contributions

D-HK and J-HK conceptualized and designed the study, analyzed the data, drafted and edited the manuscript, and approved the final submission. All authors contributed to the article and approved the submitted version.

## Conflict of Interest

The authors declare that the research was conducted in the absence of any commercial or financial relationships that could be construed as a potential conflict of interest.

## Publisher's Note

All claims expressed in this article are solely those of the authors and do not necessarily represent those of their affiliated organizations, or those of the publisher, the editors and the reviewers. Any product that may be evaluated in this article, or claim that may be made by its manufacturer, is not guaranteed or endorsed by the publisher.
