# Supplementary material for: Efficacy of Tigecycline and Linezolid Against Pan-Drug-Resistant Bacteria Isolated From Companion Dogs in South Korea
Source: Front Vet Sci. 2021 Aug 6;8:693506. doi: 10.3389/fvets.2021.693506 (PMC8377367; doi:10.3389/fvets.2021.693506)
Supplement: Supplementary file 2 [file Data_Sheet_2.pdf]

Statistical Analysis Results Report

Hanwool Knowledge Information Consulting

Highlights

1. Statistical Analysis Title

Efficacy of Tigecycline and Linezolid Against Pan-drug-resistant Bacteria Isolated from Companion Dogs in South Korea

2. Statistical analysis content

Descriptive statistics were used for the analyses of signalment, clinical data, and laboratory findings. All statistical analyses were performed using SPSS Statistics 26.0 program (IBM Corp., Armonk, NY). After cross-analysis, a chi-square test was used to compare the efficacy of LZD, TGC, and 21 different antibiotics against PDR bacteria from dogs. Differences with *P*-values < 0.05 were considered statistically significant.

3. Requester

Dong-Hyun Kim

Department of Veterinary Internal Medicine, College of Veterinary Medicine, Konkuk University, Seoul, South Korea

4. Implementing organization

company name	Hanwool Knowledge Information Consulting	Company Registration Number	105-90-97102
representative	HYUN-HO LIM	Business Contact	02-706-3695
location	Room 1311, Hanshin Building, 136-1 Mapo-dong, Mapo-gu, Seoul		

5. Date of request for analysis : 2021.06.19.

6. Statistical analysis period: 2021.06.19.~2021.06.23.

7. Submission of final report: 2021.06.23.

head of analysis: 임현호 date 2021 / 6 / 23

Hanwool Knowledge Information Consulting CEO HYUN-HO LIM

Table 3. Comparative superiority of linezolid efficacy against 21 different antibiotics to eradicate pan-drug-resistant bacteria from dogs

**Antibiotic**	***Susceptible (%)***	**Linezolid *p-value***
Amikacin	39.1%	< 0.001
Amoxycillin/Clavulanic acid	10.9%	< 0.001
Ampicillin	0.0%	< 0.001
Azithromycin	8.7%	< 0.001
Cefixime	0.0%	< 0.001
Cefotaxime	4.3%	< 0.001
Cefpodoxime	0.0%	< 0.001
Ceftazidime	9.7%	< 0.001
Cephalexin	0.0%	< 0.001
Cephazolin	15.2%	< 0.001
Ciprofloxacin	0.0%	< 0.001
Clindamycin	4.3%	< 0.001
Doxycycline	28.3%	< 0.001
Enrofloxacin	2.2%	< 0.001
Erythromycin	4.3%	< 0.001
Gentamicin	0.0%	< 0.001
Lincomycin	3.2%	< 0.001
Ofloxacin	0.0%	< 0.001
Spiramycin	2.2%	< 0.001
Sulfamethoxazole/Trimethoprim	0.0%	< 0.001
Tetracycline	0.0%	< 0.001
Linezolid (LZD)	95.7%	

Hanwool Knowledge Information Consulting

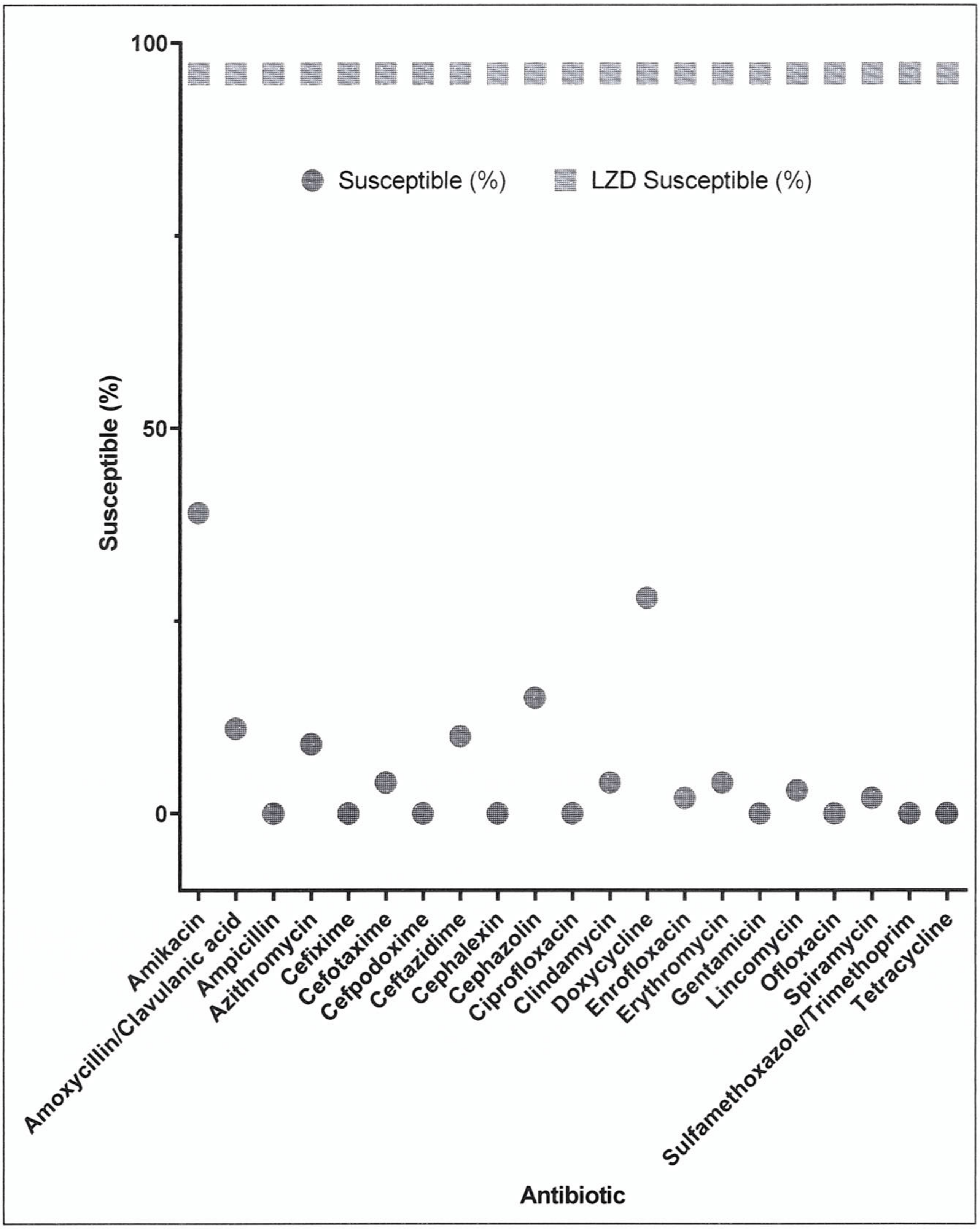
100
50
0
Susceptible (%)
LZD Susceptible (%)
Susceptible (%)
Amikacin
Amoxycillin/Clavulanic acid
Ampicillin
Azithromycin
Cefixime
Cefotaxime
Cefpodoxime
Ceftazidime
Cephalexin
Cephazolin
Ciprofloxacin
Clindamycin
Doxycycline
Enrofloxacin
Erythromycin
Gentamicin
Lincomycin
Ofloxacin
Spiramycin
Sulfamethoxazole/Trimethoprim
Tetracycline
Antibiotic

Table 4. Comparative superiority of tigecycline efficacy against 21 different antibiotics to eradicate pan-drug-resistant bacteria from dogs

Antibiotic	*Susceptible (%)*	Tigecycline *p-value*
Amikacin	40.0%	< 0.001
Amoxycillin/Clavulanic acid	11.8%	< 0.001
Ampicillin	0.0%	< 0.001
Azithromycin	7.1%	< 0.001
Cefixime	5.7%	< 0.001
Cefotaxime	7.1%	< 0.001
Cefpodoxime	1.2%	< 0.001
Ceftazidime	17.1%	< 0.001
Cephalexin	1.2%	< 0.001
Cephazolin	10.6%	< 0.001
Ciprofloxacin	1.9%	< 0.001
Clindamycin	2.4%	< 0.001
Doxycycline	18.8%	< 0.001
Enrofloxacin	2.4%	< 0.001
Erythromycin	2.4%	< 0.001
Gentamicin	2.4%	< 0.001
Lincomycin	1.9%	< 0.001
Ofloxacin	3.5%	< 0.001
Spiramycin	1.5%	< 0.001
Sulfamethoxazole/Trimethoprim	0.0%	< 0.001
Tetracycline	1.2%	< 0.001
Tigecycline (TGC)	90.6%	

Hanwool Knowledge Information Consulting

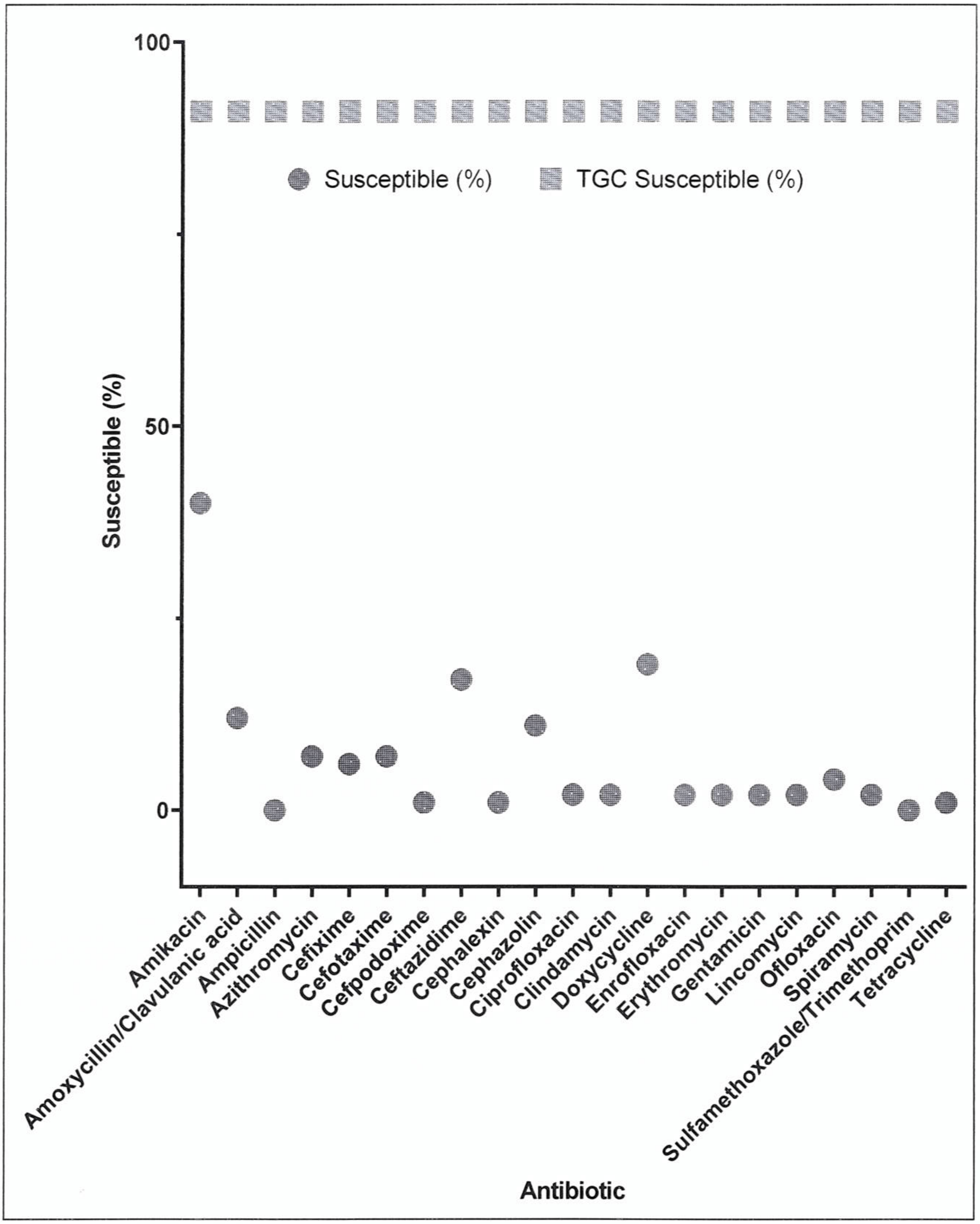
100
50
0
Susceptible (%)
Susceptible (%)
TGC Susceptible (%)
Amikacin
Amoxycillin/Clavulanic acid
Ampicillin
Azithromycin
Cefixime
Cefotaxime
Cefpodoxime
Ceftazidime
Cephalexin
Cephazolin
Ciprofloxacin
Clindamycin
Doxycycline
Enrofloxacin
Erythromycin
Gentamicin
Lincomycin
Ofloxacin
Spiramycin
Sulfamethoxazole/Trimethoprim
Tetracycline
Antibiotic
